# Development and validation of a predictive model for continuous renal replacement therapy in sepsis patients using the MIMIC-IV database

**DOI:** 10.1038/s41598-025-07647-6

**Published:** 2025-07-01

**Authors:** Binglin Song, Ping Liu, Chun Liu, Kangrui Fu, Xiangde Zheng, Ying Liu

**Affiliations:** 1 Southwest Medical University, LuZhou, 646000 China; 2 Emergency Department of Dazhou Central Hospital, Dazhou, 635000 China; 3 Clinical Medical College of North Sichuan Medical College, Nanchong, 637000 China

**Keywords:** Intensive care unit, Sepsis, Continuous renal replacement therapy, Prediction, Dynamic nomogram, Kidney diseases, Diseases, Medical research, Risk factors

## Abstract

To develop and validate a dynamic nomogram for predicting the need for continuous renal replacement therapy (CRRT) in septic patients in the intensive care unit (ICU). Data were extracted from the MIMIC-IV 3.0 database and divided into a training set and a validation set in a 7:3 ratio. Relevant risk factors were identified through LASSO regression, and a binary logistic regression model was subsequently developed. The CRRT risk nomogram was visualized using R language, with the DynNom package employed to create a dynamic nomogram. Model performance was assessed using the area under the receiver operating characteristic curve (AUC), Harrell’s C-index, and calibration curves. The clinical utility of the model was evaluated via decision curve analysis (DCA). A total of 7361 septic patients were included in this study, of which 525 required CRRT. The study identified several predictive factors for CRRT, including respiratory rate, oxygen saturation, international normalized ratio (INR), activated partial thromboplastin time (APTT), creatinine, lactate, pH, body weight, renal disease, and severe liver disease. The C-index was 0.871. The AUCs for the training and validation sets were 0.87 (95% CI: 0.8535–0.8883) and 0.86 (95% CI: 0.8282–0.8887), respectively. The calibration curves demonstrated good predictive consistency. DCA confirmed the model’s significant clinical value. The dynamic nomogram is available for visualization at: https://zhong-hua-min-zu-wan-sui.shinyapps.io/CRRT_prediction_nomogram/. We have developed a dynamic nomogram based on the MIMIC-IV database, incorporating 10 clinical features, to predict the probability of CRRT requirement in septic patients. Internal validation showed that this model exhibits robust predictive performance.

## Introduction

Acute Kidney Injury (AKI) is a common complication in critically ill patients with sepsis, often associated with poor prognosis^1^. Studies have demonstrated a close correlation between AKI and high mortality rates as well as adverse outcomes in septic patients^2^. Approximately 36–67% of critically ill patients experience AKI, which frequently leads to organ failure and death^3^. Continuous Renal Replacement Therapy (CRRT) has become the preferred treatment for patients with sepsis complicated by AKI^4^. Compared to intermittent dialysis, CRRT is more effective in removing solutes and maintaining hemodynamic stability, which is why it is widely used in Intensive Care Units (ICUs) for managing AKI patients^5,6^. Despite its efficacy, early identification of patients requiring CRRT remains a clinical challenge due to the complexities of treatment timing and patient selection^7^. In general, the decision for CRRT in septic patients may be influenced by various factors. Therefore, developing an effective predictive model is of significant importance. A review of literature databases such as PubMed reveals no existing predictive models for this scenario. In this study, we leverage the extensive clinical data provided by the MIMIC-IV database to identify easily accessible clinical indicators associated with the need for CRRT and use these indicators to construct a predictive model.

## Patient inclusion criteria

### Inclusion of subjects

We conducted a retrospective cohort study based on the MIMIC-IV database. The MIMIC-IV Database (Medical Information Mart for Intensive Care IV, Version 3.0) is an open-access, free critical care database that includes comprehensive clinical data of patients admitted to medical centers from 2008 to 2022^8,9^. As an updated version of MIMIC-III, MIMIC-IV not only integrates the latest data but also improves upon several aspects of MIMIC-II. The database records detailed information on 200,000 emergency patients and 90,000 intensive care unit (ICU) patients, covering clinical data such as demographics, vital signs, imaging results, laboratory test outcomes, and including codes from the International Classification of Diseases, 9th and 10th Editions (ICD-9 and ICD-10). Data sources comprise hourly physiological and monitoring data recorded by ICU nurses. Furthermore, all health information in MIMIC-IV has been de-identified, thus patient consent is not required^10,11^. Data extraction for this study was approved by the Institutional Review Board (IRB) at the Massachusetts Institute of Technology (MIT) (Certification No.: 65828043).

### Data extraction

We initially performed data extraction and processing using Structured Query Language (SQL) and Navicat Premium software (version 15.0.12). SQL scripts were utilized to extract patient information from the database, including sociodemographic characteristics, vital signs, laboratory parameters, complications, and microbiological data^12^. The demographic data extracted included gender, age at admission, race, weight, height, length of hospital stay, and duration of ICU stay. Vital sign data encompassed heart rate, respiratory rate, systolic blood pressure, mean arterial pressure, and oxygen saturation. Complications included hypertension, congestive heart failure, chronic pulmonary disease, diabetes, renal disease, severe liver disease, and the staging of acute kidney injury (AKI). Laboratory parameters comprised D-dimer, fibrinogen, thrombin, international normalized ratio (INR), prothrombin time (PT), partial thromboplastin time (PTT), C-reactive protein (CRP), albumin, anion gap, bicarbonate, blood urea nitrogen (BUN), creatinine, blood glucose, creatine kinase, total bilirubin, neutrophils, lymphocytes, erythrocytes and their time-related variations, leukocytes, hemoglobin, lactate, pH, platelets, N-terminal pro B-type natriuretic peptide, and troponin. In addition, we extracted the Acute Physiology and Chronic Health Evaluation (APACHE III) score and the Sequential Organ Failure Assessment (SOFA) score. All extracted variables formed the foundation for the construction of our predictive model and were subsequently utilized in predicting whether sepsis patients would require continuous renal replacement therapy (CRRT).

### Inclusion criteria

**Fig. 1:**
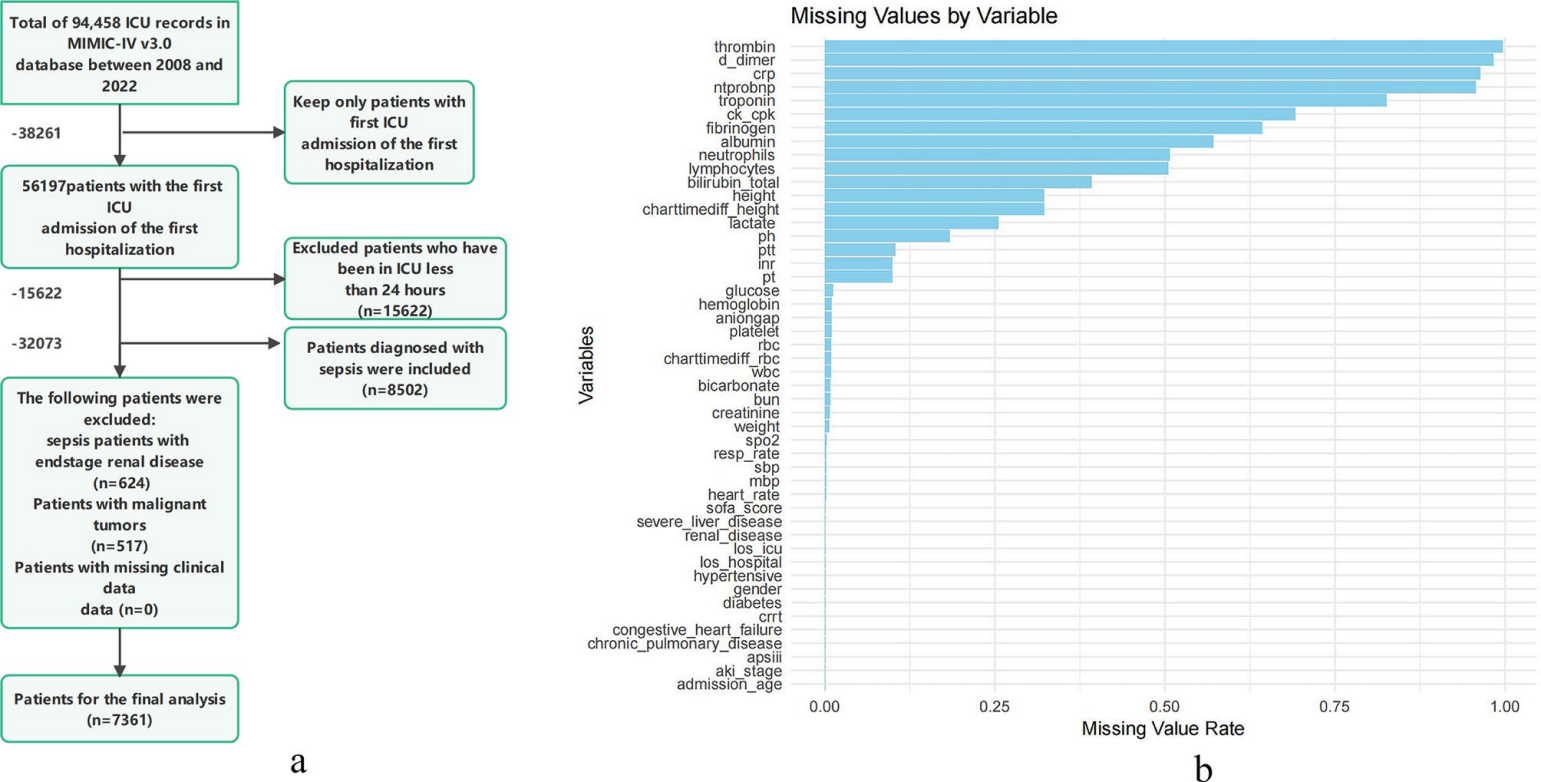
**(a)** Flow chart of data screening (Flow diagram illustrating the selection process of ICU patients from the MIMIC-IV v3.0 database (2008–2022). A total of 94,458 ICU records were screened. Patients were included if they were admitted to the ICU for the first time during their first hospitalization and stayed for at least 24 h. Further exclusions were applied for end-stage renal disease, malignancy, or missing clinical data. After these criteria, 7361 septic patients were included in the final analysis). **(b)** Missing Data Proportions of Candidate Predictors for CRRT Prediction Modeling (Missing data proportions for all candidate clinical, demographic, and laboratory variables. Variables such as thrombin, D-dimer, and CRP exhibited high missingness, with rates exceeding 90%. In contrast, core demographic variables (e.g., age, gender) and key clinical indicators (e.g., blood pressure, creatinine, comorbidities) had minimal or no missing values).

1. First-time ICU admission.
2. ICU stay > 24 h.
3. Age between 18 and 80 years.
4. Meeting the Sepsis 3.0 diagnostic criteria^13^. Sepsis patients were identified within the MIMIC-IV database using ICD-9 codes (99591, 99592, and 78552) and ICD-10 codes (R65.20, R65.21). Data from these patients were used to establish the model. The data extraction process is illustrated in Fig. 1A.

### Exclusion criteria

1. Non-septic patients.
2. Patients with end-stage renal disease.
3. Patients with malignant tumors.
4. Patients with missing clinical data.

### Outcome

In this retrospective cohort study, the outcome was defined as the requirement for continuous renal replacement therapy (CRRT) in ICU patients with sepsis.

### Statistical methods

Data analysis was conducted using SPSS version 22.0 and R version 4.4.2. Normally distributed continuous variables are expressed as mean ± standard deviation (x ± s), while non-normally distributed continuous variables are presented as median and interquartile range (M(Q1, Q3)). The Mann-Whitney U test was used to compare differences between groups for non-normally distributed data. Categorical variables are presented as count (percentage), and the Chi-square test or Fisher’s exact test was applied for categorical data. Ordinal variables were analyzed using the rank-sum test. Variables with more than 30% missing data were excluded, while those with less than 30% missing data were handled using listwise deletion, where rows with missing values were removed to ensure complete data for each variable. The missing data rates for each variable are displayed in the provided visualization (see Fig. 1B).All subjects were randomly divided into training and validation sets in a 7:3 ratio. The training set was used to build the model, and the validation set was used for model validation.

#### Univariate ROC analysis

To evaluate the predictive power of individual variables for CRRT initiation, receiver operating characteristic (ROC) curves were generated for each variable. The area under the curve (AUC) was calculated using the roc() function, and the results were visualized with the ggroc() function. This analysis enabled the identification of variables with strong discriminatory ability.

##### Correlation analysis and heatmap visualization

Pearson correlation coefficients were computed using the cor() function to assess pairwise correlations among continuous variables. The correlation matrix was visualized using the corrplot package. Statistical significance of the correlations was tested with the cor.mtest() function, and significant associations were annotated in the heatmap. This process facilitated the identification of potential multicollinearity among variables.

##### Lasso regression for variable selection

Lasso regression was applied using the glmnet() function to perform variable selection through L1 regularization. Ten-fold cross-validation was conducted with cv.glmnet() to determine the optimal penalty parameter (lambda). This approach reduced model complexity while retaining variables most predictive of CRRT requirement, thereby improving model generalizability.

##### Logistic regression modeling and nomogram construction

Multivariable logistic regression was performed using the lrm() function based on variables selected by Lasso regression. A nomogram was then constructed with the nomogram() function to provide a user-friendly tool for clinical risk estimation. Several threshold probabilities (e.g., 0.001, 0.01, 0.05) were designated as reference points to guide clinical interpretation.

##### Forest plot

A forest plot was generated using the forestploter package to display the regression coefficients (β), standard errors (SE), Wald statistics, odds ratios (OR), and their 95% confidence intervals (CI) for each covariate in the final logistic model. This visualization facilitated intuitive interpretation of effect sizes and statistical significance.

##### Multivariable ROC analysis

The overall predictive performance of the final model was assessed using multivariable ROC analysis on both the training and validation datasets. Predicted probabilities were derived from the logistic regression model, and ROC curves were plotted using the roc() and ggroc() functions. AUC values were calculated to evaluate the model’s discriminative ability across datasets.

##### Model calibration and cross-validation

Ten-fold cross-validation was conducted to evaluate the stability of the model. Calibration curves were generated using the calibrate() function with bootstrap resampling (method = “boot”). This allowed assessment of the agreement between predicted probabilities and observed outcomes, ensuring the reliability of the model in clinical settings.

##### Decision curve analysis (DCA)

Decision curve analysis was performed using the decision_curve() function to assess the clinical utility of the model at various risk thresholds. Net benefit was calculated across a range of threshold probabilities, and decision curves for the training and validation cohorts were visualized using the plot_decision_curve() function. This analysis provided insight into the model’s practical value in clinical decision-making.

## Results

### Comparison of clinical data between the two groups

Univariate analysis revealed that CRRT was significantly associated with the following variables (*P* < 0.05, as shown in Table 1): hypertension, congestive heart failure, renal disease, severe liver disease, systolic blood pressure, mean arterial pressure, respiratory rate, oxygen saturation, international normalized ratio (INR), prothrombin time, activated partial thromboplastin time, anion gap, bicarbonate, blood urea nitrogen, serum creatinine, red blood cell count, red blood cell distribution width, white blood cell count, hemoglobin, lactate, blood pH, platelet count, body weight, and heart rate.

**Table 1:** Comparison of clinical data between CRRT group and non-CRRT group [M(Q1,Q3)]

Variables	Total (*n* = 5013)	Non-CRRT group (*n* = 4488)	CRRT group (*n* = 525)	*p*
Hypertensive, n (%)				0.025
No	2879 (57)	2553 (57)	326 (62)	
Yes	2134 (43)	1935 (43)	199 (38)	
Congestive heart failure, n (%)				< 0.001
No	4130 (82)	3728 (83)	402 (77)	
Yes	883 (18)	760 (17)	123 (23)	
Chronic pulmonary disease, n (%)				1
No	4065 (81)	3639 (81)	426 (81)	
Yes	948 (19)	849 (19)	99 (19)	
Diabetes, n (%)				0.816
No	3911 (78)	3504 (78)	407 (78)	
Yes	1102 (22)	984 (22)	118 (22)	
Renal disease, n (%)				< 0.001
No	4566 (91)	4147 (92)	419 (80)	
Yes	447 (9)	341 (8)	106 (20)	
Severe liver disease, n (%)				< 0.001
No	4308 (86)	3980 (89)	328 (62)	
Yes	705 (14)	508 (11)	197 (38)	
Gender, n (%)				0.292
Feman	1838 (37)	1634 (36)	204 (39)	
Man	3175 (63)	2854 (64)	321 (61)	
Aki stage, n (%)				< 0.001
No	967 (19)	967 (22)	0 (0)	
1	932 (19)	930 (21)	2 (0)	
2	1788 (36)	1788 (40)	0 (0)	
3	1326 (26)	803 (18)	523 (100)	
Los hospital, median (Q1, Q3)	10.65 (5.76, 20.04)	9.94 (5.67, 18.48)	20.93 (9.95, 36.09)	< 0.001
Admission age, median (Q1, Q3)	50.21 (40.3, 55.81)	50.41 (40.31, 55.87)	49.09 (39.9, 55.33)	0.243
Los icu, median (Q1, Q3)	4.05 (2.08, 9.13)	3.71 (1.97, 7.87)	11.64 (5.11, 19.88)	< 0.001
Sofa score, median (Q1, Q3)	3 (2, 5)	3 (2, 4)	6 (4, 9)	< 0.001
Sbp, median (Q1, Q3)	118 (104, 134)	118 (104, 135)	113 (98, 131)	< 0.001
Mbp, median (Q1, Q3)	82 (72, 94)	83 (72, 95)	79 (68, 91)	< 0.001
Resp rate, median (Q1, Q3)	19 (16, 24)	19 (15, 24)	22 (18, 27)	< 0.001
spo2, median (Q1, Q3)	99 (96, 100)	99 (96, 100)	97 (93, 100)	< 0.001
inr, median (Q1, Q3)	1.3 (1.2, 1.6)	1.3 (1.1, 1.5)	1.6 (1.3, 2.4)	< 0.001
pt, median (Q1, Q3)	14.5 (12.8, 17.4)	14.3 (12.7, 16.8)	17.8 (14.1, 25.9)	< 0.001
ptt, median (Q1, Q3)	30.8 (26.9, 38)	30.3 (26.7, 36.4)	39.6 (31.1, 52.8)	< 0.001
Aniongap, median (Q1, Q3)	14 (11, 17)	14 (11, 17)	18 (15, 23)	< 0.001
Bicarbonate, median (Q1, Q3)	22 (19, 25)	22 (19, 25)	19 (15, 23)	< 0.001
Bun, median (Q1, Q3)	16 (12, 26)	16 (11, 23)	31 (19, 53)	< 0.001
Creatinine, median (Q1, Q3)	1 (0.7, 1.4)	0.9 (0.7, 1.3)	2.2 (1.4, 3.9)	< 0.001
Glucose, median (Q1, Q3)	128 (106, 167)	128 (106.75, 165)	133 (103, 194)	0.141
rbc, median (Q1, Q3)	3.62 (3.04, 4.24)	3.66 (3.08, 4.25)	3.29 (2.6, 4.07)	< 0.001
wbc, median (Q1, Q3)	12.7 (8.7, 17.5)	12.6 (8.8, 17.4)	13.6 (8.2, 19.3)	0.048
Hemoglobin, median (Q1, Q3)	10.8 (9.1, 12.7)	10.9 (9.3, 12.7)	10 (8.2, 12.1)	< 0.001
Lactate, median (Q1, Q3)	1.8 (1.2, 2.9)	1.7 (1.2, 2.6)	2.8 (1.7, 5.6)	< 0.001
ph, median (Q1, Q3)	7.36 (7.29, 7.42)	7.37 (7.3, 7.42)	7.3 (7.2, 7.38)	< 0.001
Platelet, median (Q1, Q3)	180 (124, 248)	184 (129, 252)	137 (86, 214)	< 0.001
apsiii, median (Q1, Q3)	46 (32, 66)	43 (31, 60)	82 (65, 99)	< 0.001
Weight, median (Q1, Q3)	85 (71.2, 100.9)	84 (70.6, 100)	93 (76, 111)	< 0.001
heart_rate, median (Q1, Q3)	93 (80, 108)	92 (80, 107)	100 (85, 117)	< 0.001

Baseline clinical characteristics of patients receiving continuous renal replacement therapy (CRRT) versus those not receiving CRRT. Categorical variables are presented as counts (percentages), and continuous variables are expressed as medians with interquartile ranges [M (Q1, Q3)]. Statistically significant differences were observed in variables such as AKI stage, renal disease, severe liver disease, and ICU length of stay. P-values were calculated using the chi-square test for categorical variables and the Mann–Whitney U test for continuous variables.

### ROC analysis

**Fig. 2:**
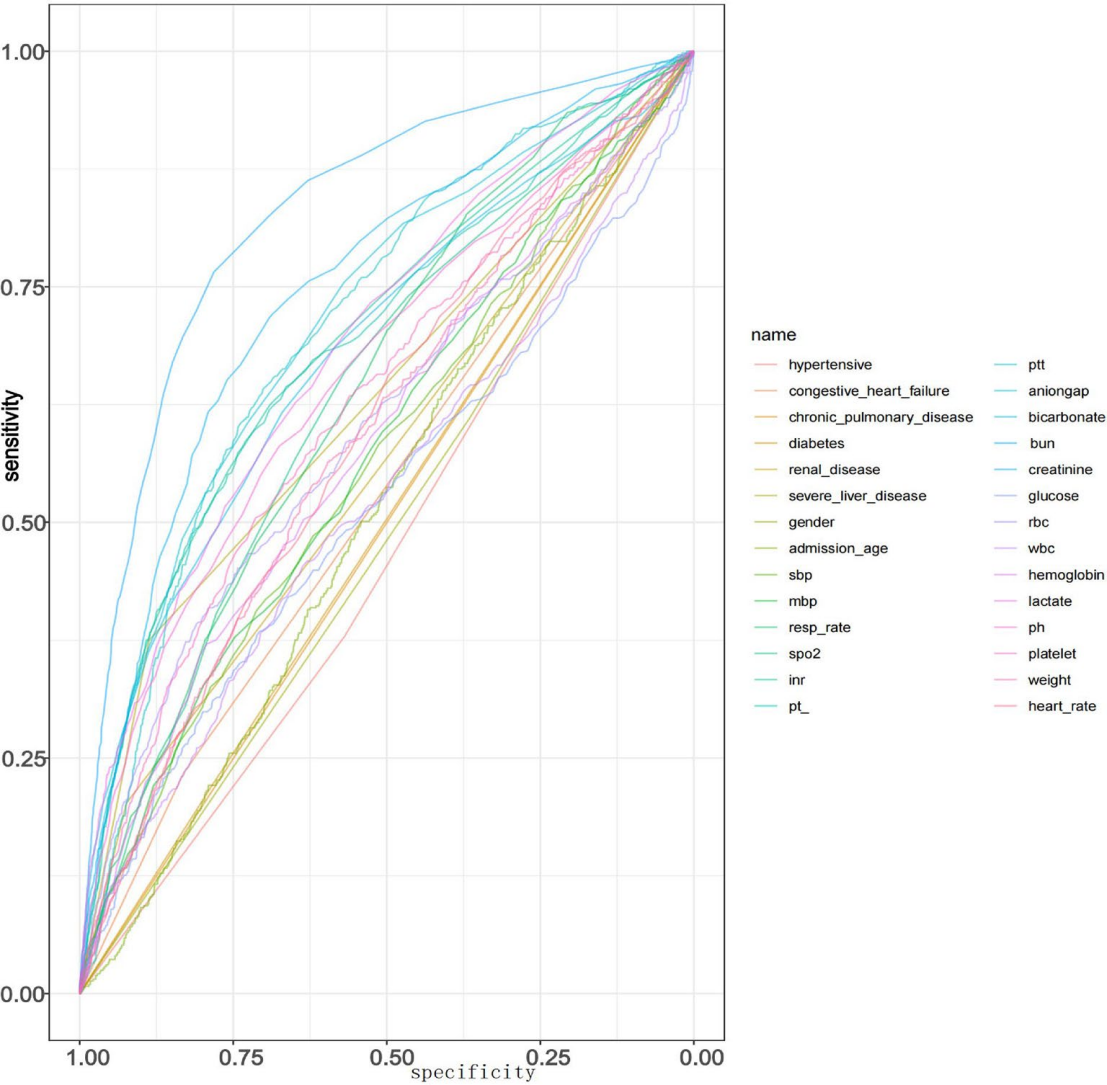
ROC curves of individual predictors for CRRT requirement. (Receiver operating characteristic (ROC) curves for individual clinical and laboratory variables in predicting the requirement for continuous renal replacement therapy (CRRT). Each curve represents the discriminatory performance of a single variable, with sensitivity plotted against 1-specificity. Variables such as *PT*, *INR*, *creatinine*, and *spo2* demonstrated relatively higher predictive performance compared to others. The proximity of most curves to the diagonal suggests that individual predictors alone may have limited discriminative power).

**Fig. 3:**
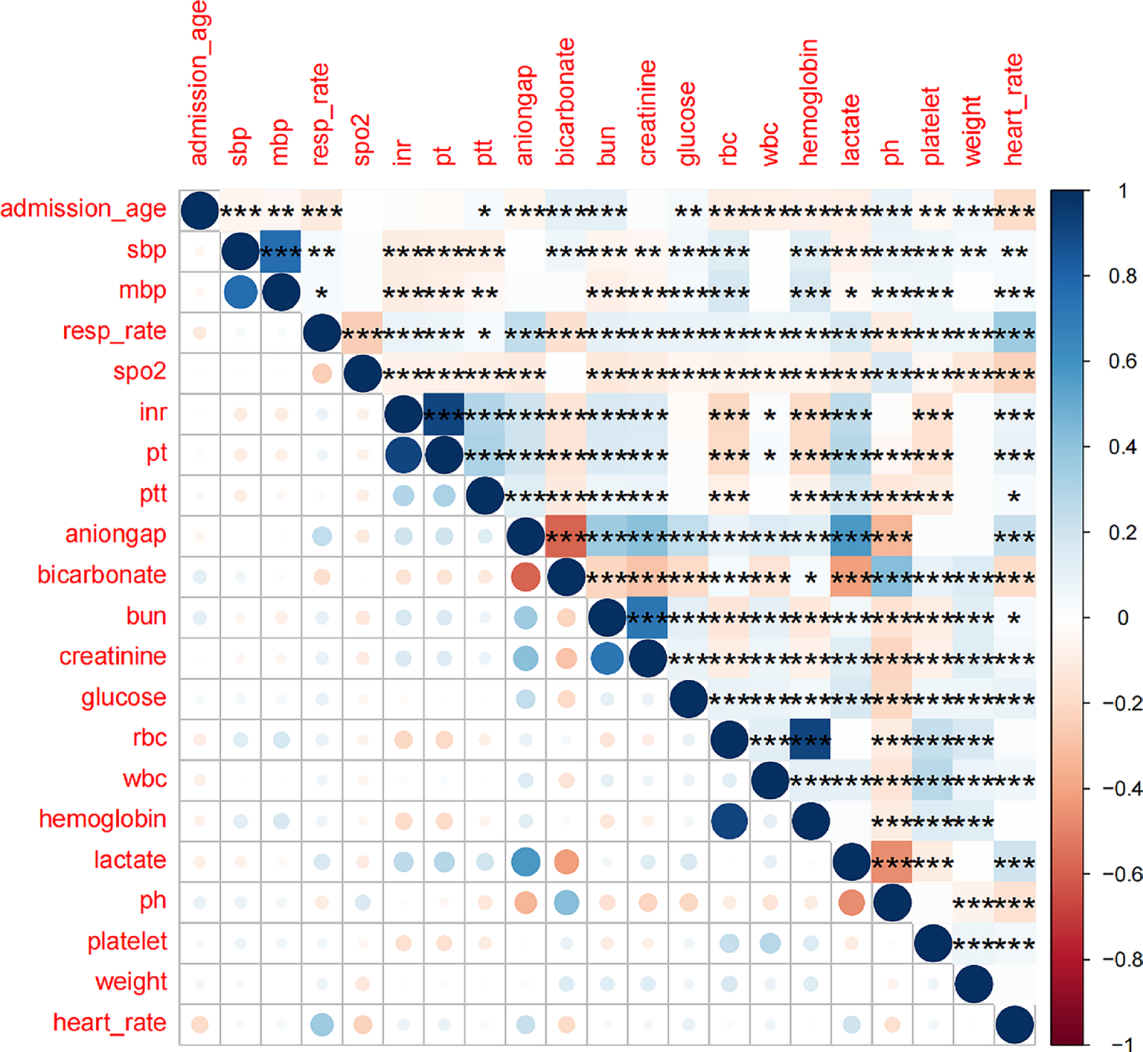
Correlation Matrix of Key Predictive Variables(Correlation matrix displaying the pairwise Pearson correlations among variables used in the predictive modeling. Color intensity and circle size represent the strength and direction of correlation. Blue indicates positive and red indicates negative correlations. Statistical significance is marked with asterisks (*p < 0.05; **p < 0.01; ***p < 0.001). The figure includes both continuous and binary variables, the latter encoded as 0 and 1 for analysis).

The ROC curves for each variable were calculated, and the corresponding AUC values were extracted. A correlation heatmap was also generated (Figs. 2 and 3).

### Preliminary screening of CRRT risk factors using Lasso regression

**Fig. 4:**
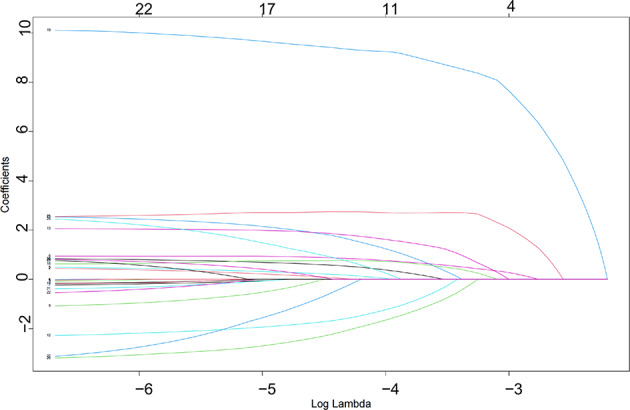
LASSO coefficient profiles of candidate predictors (Coefficient profiles of candidate predictors plotted against the log-transformed lambda values in the LASSO regression. Each curve represents the coefficient trajectory of a single variable. As the regularization parameter increases, more coefficients shrink toward zero, indicating variable exclusion. The numbers shown at the top of the plot correspond to the number of non-zero coefficients at each value of lambda).

**Fig. 5:**
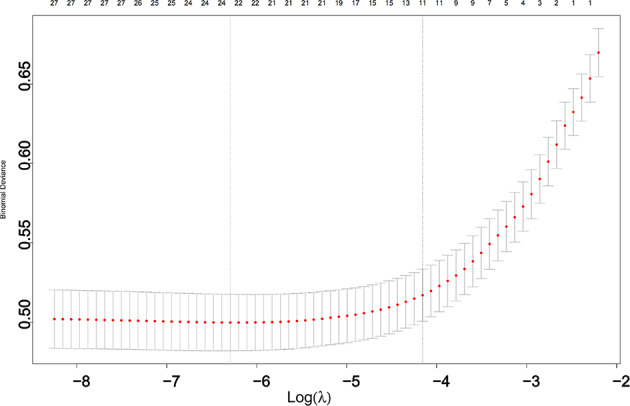
Cross-validation curve for selecting the optimal lambda in LASSO regression (Ten-fold cross-validation results for tuning the regularization parameter (λ) in the LASSO regression. The horizontal axis shows the log-transformed values of λ, and the vertical axis indicates the corresponding mean cross-validated error. Red dots represent the average error at each λ, with grey error bars showing the standard deviation. The number of non-zero coefficients at each λ is indicated at the top of the plot.)

To avoid multicollinearity in the data analysis, Lasso regression was applied to select variables with a univariate P-value < 0.05. As the λ value increased, the penalty was intensified, and the regression coefficients of the model’s independent variables were progressively shrunk to zero. Ultimately, 11 variables with non-zero coefficients were selected, including: renal disease, severe liver disease, respiratory rate, oxygen saturation, international normalized ratio (INR), activated partial thromboplastin time, blood urea nitrogen, serum creatinine, lactate, blood pH, and body weight (Figs. 4 and 5).

### Logistic regression analysis of CRRT risk factors

**Fig. 6:**
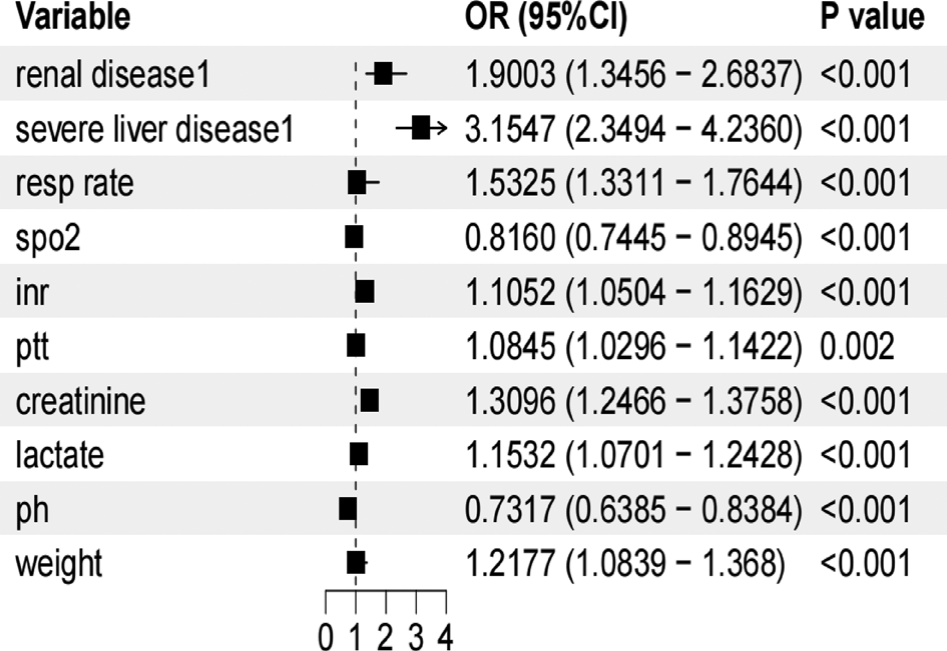
Forest plot of multivariable logistic regression for CRRT prediction (Forest plot displaying the odds ratios (OR), 95% confidence intervals (CI), and p-values from the multivariable logistic regression analysis. Variables such as severe liver disease, renal disease, and respiratory rate were associated with a significantly increased risk of requiring CRRT. OR values are visually represented by squares, and horizontal lines indicate 95% CI. P-values < 0.05 denote statistical significance.)

A binary logistic regression analysis was conducted with the decision to perform CRRT as the dependent variable and the feature variables selected by Lasso and stepwise regression as independent variables. Ten predictive factors were ultimately identified for the model, which included: renal disease, severe liver disease, respiratory rate, oxygen saturation, international normalized ratio (INR), activated partial thromboplastin time, serum creatinine, lactate, blood pH, and body weight (see Table 2).The forest plot visually presents the regression results and the range of confidence intervals (Fig. 6).

**Table 2:** Logistic regression analysis of independent risk factors for CRRT.

Factor	β	S.E.	z	*p*	OR (95% CI)
Resp rate	0.426	0.071	5.938	< 0.0001	1.532 (1.331–1.764)
spo2	− 0.203	0.046	− 4.340	< 0.0001	0.816 (0.744–0.894)
Inr	0.100	0.025	3.851	0.0001	1.105 (1.050–1.163)
Ptt	0.081	0.026	3.063	0.0022	1.085 (1.030–1.142)
Creatinine	0.269	0.025	10.722	< 0.0001	1.310 (1.247–1.376)
Lactate	0.142	0.038	3.735	0.0002	1.153 (1.070–1.243)
Ph	− 0.312	0.069	− 4.498	< 0.0001	0.732 (0.639–0.838)
Weight	1.196	0.059	3.315	0.0009	1.218 (1.084–1.368)
Renal disease − 1:0	0.642	0.176	3.645	0.0003	1.900 (1.346–2.684)
Severe liver disease − 1:0	1.148	0.150	7.639	< 0.0001	3.155 (2.349–4.236)

Results of the multivariable logistic regression identifying independent predictors of continuous renal replacement therapy (CRRT) requirement. Variables such as elevated respiratory rate, creatinine, lactate, and the presence of renal or severe liver disease were significantly associated with increased odds of CRRT initiation. In contrast, higher *spo2* and *ph* values were associated with reduced risk. Odds ratios (OR) and 95% confidence intervals (CI) are provided alongside corresponding regression coefficients (β), standard errors (SE), z-scores, and p-values.

### Model performance evaluation

**Fig. 7:**
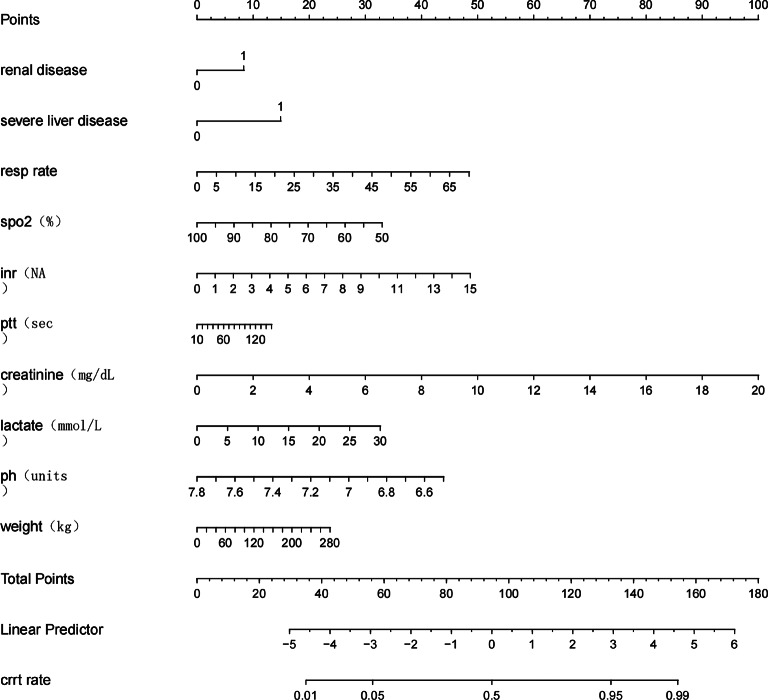
Nomogram for predicting the risk of CRRT requirement (Nomogram developed from a multivariable logistic regression model to predict the probability of requiring continuous renal replacement therapy (CRRT). Each predictor corresponds to a point scale at the top. To estimate a patient’s CRRT risk, locate each variable value on its corresponding axis, draw a vertical line upward to the “Points” scale to assign a score, then sum the total points and map them to the “CRRT rate” scale at the bottom. The nomogram facilitates individualized risk estimation at the bedside.)

**Fig. 8:**
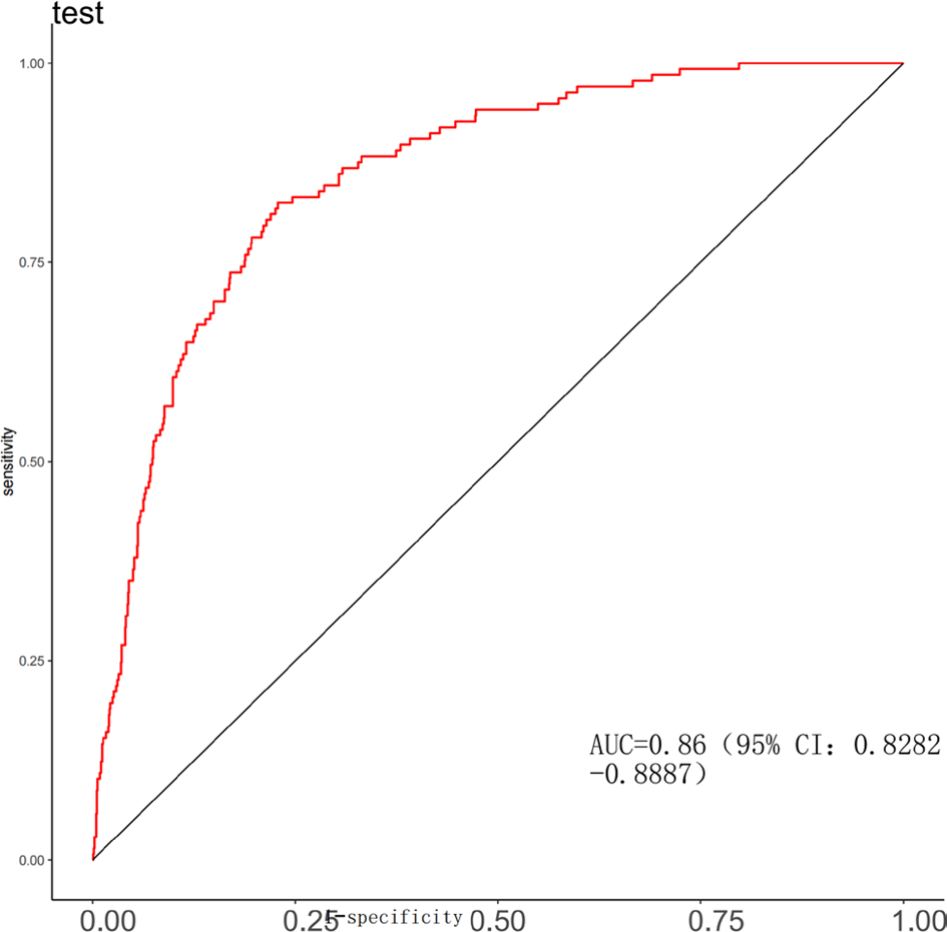
Receiver operating characteristic (ROC) curve for the training set (ROC curve showing the discrimination performance of the predictive model in the training dataset. The model achieved an area under the curve (AUC) of 0.87 (95% CI: 0.8535–0.8883), indicating excellent ability to distinguish between patients who required CRRT and those who did not.)s.

**Fig. 9:**
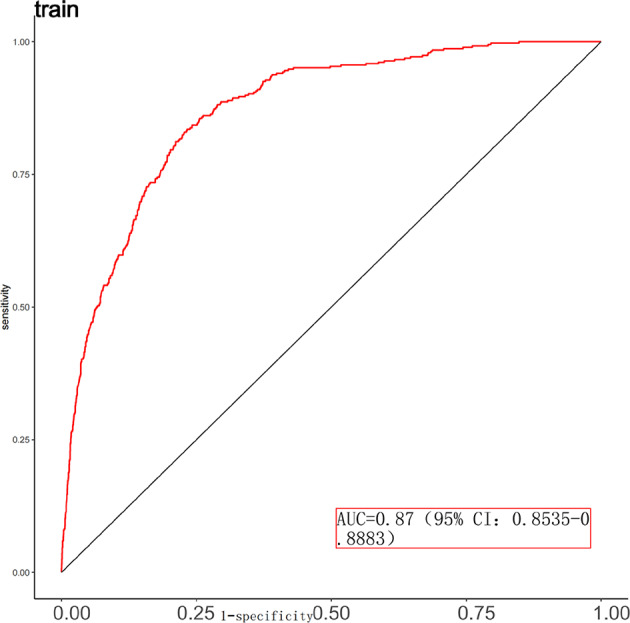
Receiver operating characteristic (ROC) curve for the validation set. (ROC curve illustrating the discrimination performance of the predictive model in the validation dataset. The area under the curve (AUC) was 0.87 (95% CI: 0.8535–0.8883), indicating that the model maintained strong predictive accuracy in an independent dataset, consistent with its performance in the training set.)

**Fig. 10:**
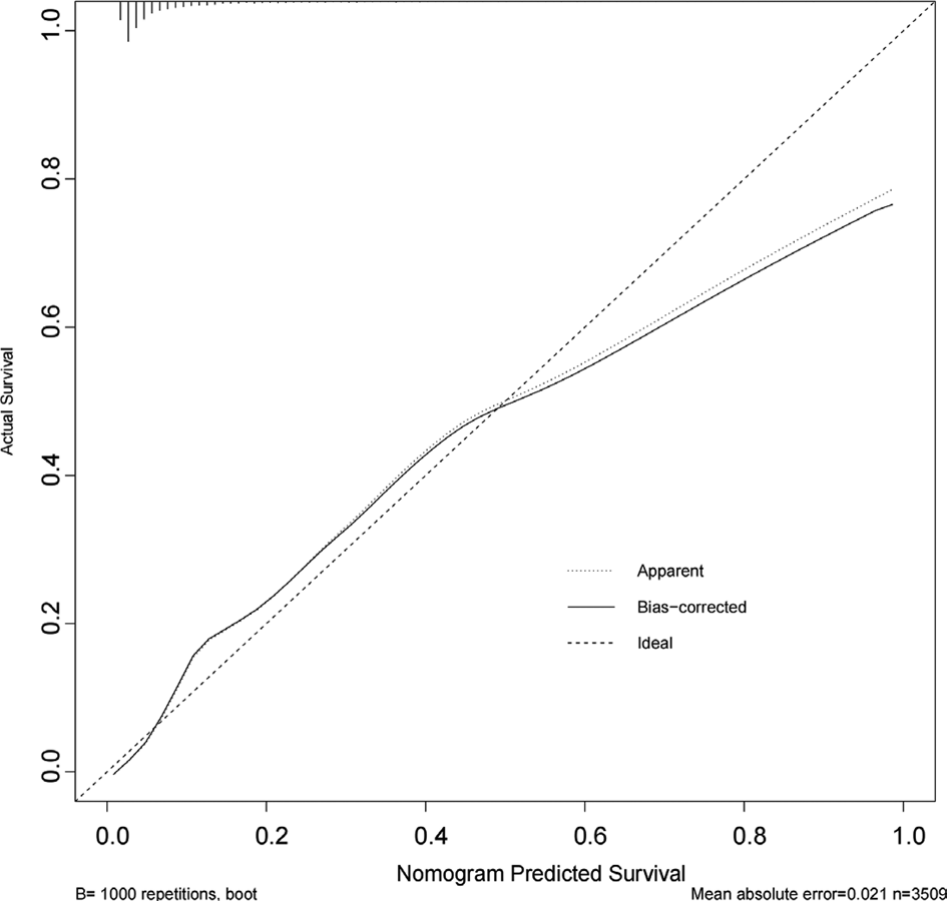
Calibration curve for the training set. Calibration curve of the predictive model in the training dataset. The plot compares the predicted probabilities of CRRT with the actual outcomes. The solid line represents the bias-corrected performance estimated via 1000 bootstrap resamples, while the dotted line indicates the ideal calibration (perfect agreement between predicted and observed outcomes). The mean absolute error was 0.021, suggesting excellent agreement and minimal deviation from the ideal line.

**Fig. 11:**
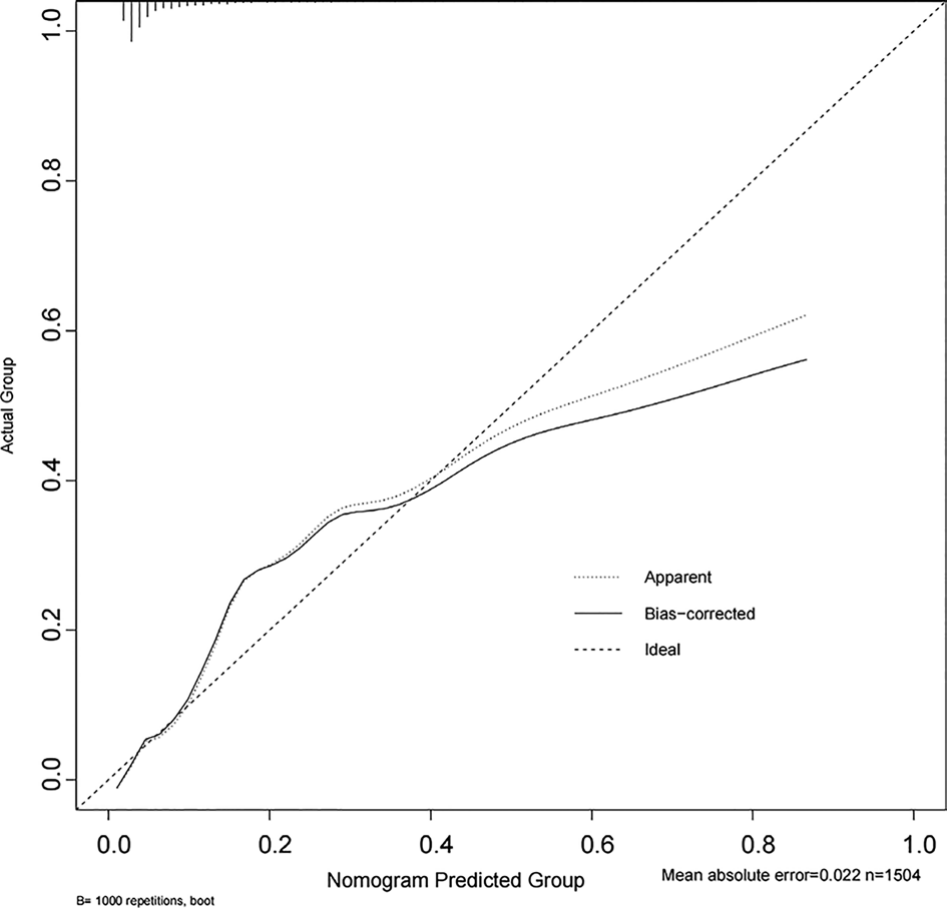
Calibration curve for the validation set. Calibration curve of the predictive model in the validation dataset. The solid line represents the bias-corrected performance based on 1000 bootstrap resamples, while the dashed line indicates perfect calibration (ideal reference). The model’s predictions showed good agreement with observed outcomes across risk strata. The mean absolute error was 0.022, suggesting satisfactory calibration performance in the external validation set.

**Fig. 12:**
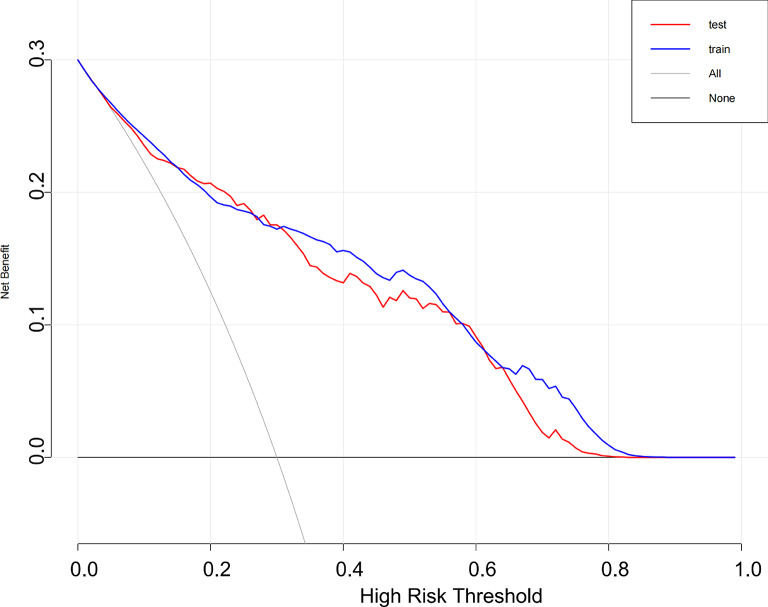
Decision curve analysis of the predictive model. (Decision curve analysis (DCA) showing the net clinical benefit of the predictive model across a range of threshold probabilities in both the training (blue) and validation (red) datasets. The gray lines represent the default strategies of treating all patients (“All”) and treating none (“None”). The model provided greater net benefit than either default strategy when the threshold probability was between approximately 0.05 and 0.65, demonstrating its potential utility in guiding clinical decision-making regarding CRRT initiation).

**Fig. 13:**
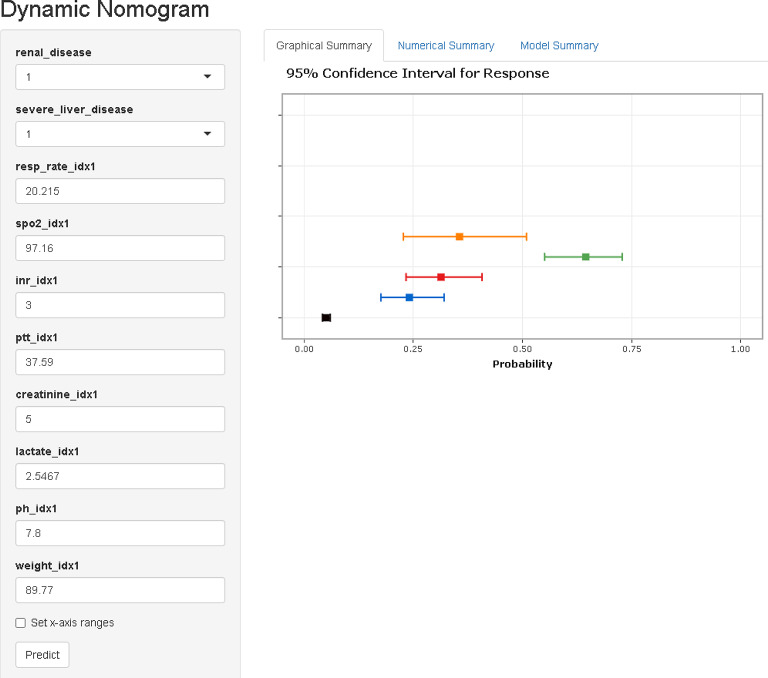
Web-based dynamic nomogram for predicting CRRT requirement. Screenshot of the online dynamic nomogram (available at: https://zhong-hua-min-zu-wan-sui.shinyapps.io/CRRT_prediction_nomogram/) for individualized prediction of CRRT requirement. Users can input patient-specific values for key predictors, including renal function, liver status, vital signs, and laboratory parameters. The graphical output presents the predicted probability along with the 95% confidence interval. This tool allows for real-time, user-friendly risk estimation at the bedside or in clinical decision-making systems.

A predictive model was constructed using binary logistic regression, and a Nomogram was plotted based on this model to assign scores to various parameters, thereby quantifying the probability of undergoing Continuous Renal Replacement Therapy (CRRT) (Fig. 7). The model demonstrated outstanding predictive performance, with a C-index of 0.87, indicating high accuracy in distinguishing between CRRT and non-CRRT patients. The areas under the curve (AUC) for the training and validation sets were 0.87 (95% CI: 0.8535–0.8883) and 0.86 (95% CI: 0.8282–0.8887), respectively, suggesting stable and robust performance across different datasets (Figs. 8 and 9). The calibration curves show a high degree of consistency between the model’s predicted values and the actual observed values (Figs. 10 and 11). The Brier scores were calculated, with a value of 0.00101 for the training set and 0.00166 for the test set, indicating slight deviations in the low-risk (0–0.2) and high-risk (0.8–1.0) intervals. In the moderate-risk range (0.2–0.8), the model’s predictions were more accurate. Additionally, decision curve analysis (DCA) demonstrated that the model provided significant net benefits across a range of threshold values, further supporting its clinical utility in real-world settings and offering valuable guidance for clinical decision-making (Fig. 12). Furthermore, we developed an online prediction system (https://zhong-hua-min-zu-wan-sui.shinyapps.io/CRRT_prediction_nomogram/) designed to provide a more accessible tool for clinicians in daily practice. This system facilitates the real-time assessment of whether septic patients meet the criteria for CRRT, thus enabling early intervention in clinical settings (Fig. 13).

## Discussion

In septic patients, acute liver and kidney failure are common complications, often necessitating continuous renal replacement therapy (CRRT) to correct multi-organ dysfunction. The systemic inflammatory response induced by sepsis frequently results in reduced renal blood flow, tubular damage, and metabolic disorders, thereby exacerbating renal failure.

CRRT not only eliminates harmful substances, metabolic byproducts, and toxins accumulated in the body, but also maintains acid-base balance and electrolyte stability, facilitating the restoration of homeostasis and supporting organ function recovery in septic patients^14^. Furthermore, CRRT reduces the systemic load, alleviating cardiac burden, particularly in elderly patients, and effectively lowers the incidence of heart failure, thereby further reducing in-hospital mortality^15^. Consequently, identifying high-risk factors for CRRT in septic patients is crucial for the early identification of at-risk individuals, reducing adverse outcomes, and guiding clinical treatment. Previous studies have demonstrated that prediction models developed using nomograms are highly regarded for their simplicity and intuitive nature^16^. Based on the clinical characteristics of septic patients, this study emphasizes easily accessible clinical variables to construct a nomogram model incorporating ten factors: renal disease, severe liver disease, respiratory rate, oxygen saturation, INR, PTT, creatinine, lactate, pH, and body weight. This model predicts the necessity of CRRT in septic patients and provides clinicians with valuable guidance for clinical decision-making.

Our study reveals that septic patients with severe liver disease are 3.16 times more likely to require CRRT compared to those without liver disease (Table 2). Severe liver injury is an important predictor of CRRT in septic patients^17^. This finding aligns with previous research, as severe liver injury often leads to liver failure, with pathophysiological mechanisms including excessive activation of inflammatory responses, impaired detoxification, and coagulation dysfunction. These factors collectively exacerbate sepsis-associated renal injury^18^. When liver function is compromised, levels of endotoxins and inflammatory mediators are significantly elevated, further promoting the development of multiple organ dysfunction syndrome (MODS) and increasing the risk of CRRT in septic patients^19^.

Our study found that elevated serum creatinine levels and kidney dysfunction at admission significantly increase the likelihood of requiring CRRT in septic patients^20^. Specifically, our results indicate that the odds of requiring CRRT are 1.9 times higher for patients with renal dysfunction than for those without (Table 2). This finding is consistent with prior research, as elevated serum creatinine is an independent risk factor for CRRT in sepsis patients^21^. Furthermore, chronic kidney disease (CKD) or a history of acute kidney injury (AKI) in septic patients amplifies their risk of systemic inflammatory response syndrome (SIRS), which can progress to sepsis-induced AKI^22,23^. The compromised filtration and excretory capabilities in patients with underlying renal disease, coupled with metabolic disturbances and susceptibility to toxic injuries, further contribute to the increased demand for CRRT^24^.

Sepsis-induced metabolic abnormalities can lead to impaired oxidative phosphorylation, heightened inflammatory responses, and progressive organ dysfunction^23^. CRRT plays a crucial role in rapidly eliminating metabolic acids and inflammatory mediators, thereby improving systemic homeostasis^24–26^. Additionally, serum lactate levels were identified as a critical indicator in septic patients, reflecting the severity of tissue hypoxia and serving as a key determinant for initiating CRRT. Integrating lactate levels into predictive models could enhance clinicians’ ability to identify patients requiring CRRT and optimize therapeutic strategies^25^.

Elevated respiratory rates (“tachypnea”) are a common manifestation of acute respiratory distress caused by sepsis. This compensatory mechanism suggests the onset of systemic hypoxia^27,28^. The relationship between sepsis and acute respiratory distress syndrome (ARDS) is well-documented, as ARDS may result from sepsis-induced pulmonary injury or systemic hypoxia. Blood gas abnormalities, such as reduced PaO₂ and elevated respiratory rates, exacerbate tissue hypoxia and organ dysfunction, further underscoring the necessity of CRRT in these patients. Rocchwerker et al. demonstrated that septic patients requiring mechanical ventilation with oxygen saturation levels below 90% had a significantly higher risk of developing MODS^27^. Similarly, our results indicate that for every 1-unit increase in respiratory rate, the odds of requiring CRRT increase by 53%. In summary, early identification of respiratory rate abnormalities, combined with metabolic and blood gas assessments, can improve predictive accuracy for initiating CRRT and enhance clinical outcomes.

INR (international normalized ratio) and PTT (partial thromboplastin time) are important predictors for determining the need for continuous renal replacement therapy (CRRT) in septic patients^29,30^. Abnormalities in INR and PTT reflect impaired coagulation function and compromised renal-hepatic axis function in septic patients, highlighting their critical role in assessing the necessity of CRRT^31,32^.

Body weight serves as a key factor in assessing volume load and intravascular status. Excessive weight often correlates with an increased risk of fluid overload and impaired hemodynamic stability^33,34^. Previous studies have demonstrated that volume overload is closely associated with adverse outcomes, especially in critically ill patients in the intensive care unit (ICU)^29,35^. In such contexts, CRRT plays a vital role in correcting fluid imbalance, facilitating ultra-filtration to remove excess fluid and toxins, and maintaining hemodynamic stability. Furthermore, body weight can indirectly indicate nutritional status and the severity of comorbidities such as malnutrition or cachexia^36^. In patients with these conditions, CRRT becomes essential for stabilizing homeostasis and supporting survival.

Traditionally, clinical evaluation of the need for CRRT relied on markers of organ dysfunction and clinical judgment, such as acute kidney injury (AKI) after ICU admission, severe acid-base imbalances (e.g., metabolic acidosis), or refractory fluid overload. However, these criteria are subjective and may vary between clinicians, making it challenging to predict the need for CRRT early and consistently in septic patients. Developing standardized tools to identify high-risk individuals with greater accuracy could significantly enhance early intervention and improve outcomes.

This study innovatively utilized routinely collected physiological and laboratory data from patients, including renal disease, severe liver disease, respiratory rate, oxygen saturation, international normalized ratio (INR), partial thromboplastin time (PTT), creatinine, lactate, blood pH, and body weight. Based on the MIMIC-IV database and logistic regression analysis, a predictive model was developed and validated. This model incorporates feature selection and optimization, offering a more objective and precise evaluation compared to traditional methods. By reducing the impact of subjective judgment, it significantly improves predictive accuracy.

Additionally, the proposed predictive model is highly adaptable to real-world clinical applications, particularly in resource-limited healthcare settings. As it allows for immediate evaluation using routinely available patient data upon hospital admission, the model provides scientific insights for early identification and risk stratification of high-risk septic patients. It fills the gap in existing predictive models for this population and offers a valuable reference for early intervention and clinical decision-making.

## Conclusion

This study successfully developed a predictive model based on the MIMIC-IV database to determine whether septic patients require continuous renal replacement therapy (CRRT). The model utilizes readily available indicators upon hospital admission, providing clinicians with a simple and objective tool to assist in predicting the need for CRRT.

The strength of this study lies in its use of extensive real-world data from the MIMIC-IV database, which enhances the reliability of the findings. Furthermore, the model demonstrated excellent predictive performance, highlighting its significant potential for early prediction of CRRT needs in clinical settings.

### Limitations

Firstly, the model was developed using data from ICU septic patients in the MIMIC-IV database, validated through a dataset split (training and validation sets), without using external datasets (e.g., data from other ICU databases or hospitals) for external validation. This may introduce population-specific biases and limit the model’s generalizability. We acknowledge this limitation and recommend that future studies incorporate external validation. Further validation through multicenter trials is crucial for expanding the model’s applicability.

Secondly, although the model performs well, some variables have missing data, which may affect the prediction accuracy. Fluid balance and urine output were not included in the model because the scope was defined during the design phase to focus on vital signs, laboratory indicators, and complications to ensure better operability and generalizability. Future studies will include additional relevant variables.

Furthermore, although the nomogram is accessible online, we have not yet conducted usability testing in real-world clinical environments. As a result, its findings may not fully represent its usage in diverse healthcare settings or conditions. We plan to conduct usability testing in clinical environments in future studies to further validate its applicability and real-world effectiveness.

## Data Availability

The dynamic nomogram is available for visualization at: https://zhong-hua-min-zu-wan-sui.shinyapps.io/CRRT_prediction_nomogram/.
